# An Independent Organisation

**Published:** 1914-03-21

**Authors:** 


### AN INDEPENDENT ORGANISATION.

The Doctors' Wives Association, the aim of which will be to organise a free service and purchasing agency, will be separately organised. All that we can offer is free service and influence to secure to doctors' wives who join the new Association the maximum facilities for obtaining the advantages which have been indicated in the foregoing pages. The difficulties and resulting losses to which wives of medical men are subjected, that have been brought to our notice with a request that we may consider the whole question and suggest a remedy, prove to demonstration that the proposed Association could render material aid and benefit to all who join it, providing the original or pioneer members amount to at least 1,000 by the middle of April. The first essential act of co-operation, therefore, is that the more enterprising of the doctors' wives should sign the coupon and post it without delay. The first thousand signatures should undoubtedly occupy an honoured position by taking this step, and it is hoped that it may be possible to mark their independence of spirit and enterprise when the new Association has proved a success. All communications should be addressed to "Co-operation," c/o The Scientific Press, Ltd., 28 & 29 Southampton Street, Strand, London, W.C.

